# Reducing pain using vibrating device during local anesthesia among Indian pediatric dental patients

**DOI:** 10.6026/973206300200781

**Published:** 2024-07-31

**Authors:** Aanchal Banka, Ruchi Gulati, Durga R, Ashtha Arya, Abarna P, Shemna MK, Pratik Surana

**Affiliations:** 1 Department of Conservative Dentistry and Endodontics, Kalinga Institute of Dental Sciences, KIIT Deemed to be University, Bhubaneswar, Odisha-751024, India; 2 Department of Dentistry, Atal Bihari Vajpayee Government Medical College, Vidisha, Madhya Pradesh, India; 3 Department of Dentistry, All India Institute of Medical Sciences, Gorakhpur, Uttar Pradesh, India; 4 Department of Conservative Dentistry and Endodontics, SGT Dental College, Hospital and Research Institute, SGT University, Gurugram, Haryana- 122505, India; 5 Consultant Orthodontist, Nagercoil, Tamilnadu, India; 6 Department of Pediatric and Preventive dentistry, KMCT Dental College, Kozhikode, Kerala, India; 7 Department of Periodontics and Preventive Dentistry, Maitri College of Dentistry and Research Centre, Durg, Chhattisgarh, India

**Keywords:** Vibrating Device, Buzzy System, Pain

## Abstract

The administration of local anesthesia constitutes one of the most anxiety-inducing and painful stimuli in pediatric dentistry. Therefore, it is of interest to evaluate the effectiveness of vibrating device in comparison to the conventional method for mitigating discomfort while administration of local anesthesia. A total of 30 children aged between 6 and 10 years, requiring local anesthesia for routine dental treatment, were allocated into two groups: a control group and an experimental group, with 15 children in each cohort. In the experimental group, a vibrating device was concurrently placed over the cheek during the administration of the local anesthesia. Pain and discomfort were assessed using both the Wong-Baker FACES Pain Rating Scale (WBFPRS) and the FLACC (Face, Legs, Activity, Cry, CONSOL ability) scale. It was observed that use of the vibrating device was found to be effective in reducing pain and discomfort during the administration of intraoral local anesthesia.

## Background:

Administering local anesthesia to children often causes significant anxiety and discomfort, making the process challenging for both the patient and the practitioner. Traditional methods often fail to alleviate the pain and fear associated with needle injections. The use of vibrating devices has emerged as a potential solution, aiming to minimize discomfort during medical procedures by providing a distracting stimulus [1]. This clinical study investigates the efficiency of a vibrating device in lessening the pain and discomfort whiles the administration of local anesthesia in children. By offering a novel method to ease pediatric patients' anxiety and improve their overall experience, this research hopes to contribute to more effective and compassionate healthcare practices [2].

The study focuses on evaluating the perceived pain, anxiety levels, and overall satisfaction among young patients undergoing local anesthesia treatments with and without the use of a vibrating device. Parent and practitioner feedback are also analyzed to gain a comprehensive understanding of the device's impact [3]. Since the development of the Buzzy® device, there has been a notable scarcity of research investigating its effectiveness in administering local anesthesia for dental procedures in pediatric populations [3,4]. Therefore, it is of interest to determine whether this non-invasive, cost-effective tool can significantly enhance patient comfort, thereby potentially improving cooperation and outcomes in pediatric medical care.

## Materials and Methods:

A total of 30 children, aged between 6 and 10 years, participated in this study. The inclusion criteria for the study are as follows: only children who do not have systemic illnesses or allergies are eligible. Additionally, these children must demonstrate cooperativeness and require infiltration local anesthesia for dental procedures. It is imperative that appropriate parental consent is obtained for participation. Conversely, the exclusion criteria specify that children diagnosed with systemic diseases are not eligible for the study. Children who exhibit behavioral management difficulties or have known allergies to local anesthetic agents are also excluded. Finally, children under the age of six years will not be considered for this study. Selected subjects were arbitrarily assigned into two distinct groups: a control group (n=15) and an experimental group (n=15).

## Group I control group:

A conventional 2-mL syringe (DispoVan, Hindustan Syringes and Medical Devices Ltd., New Delhi) is employed to administer a local anesthetic (LOX 2% adrenaline, Neon Laboratories Ltd., Mumbai) to the region adjacent to the tooth necessitating an invasive treatment procedure, without additional interventions.

## Group II experimental group:

The child was positioned in the dental chair, and explanation of the device was given in simple language before permitting the child to interact with Buzzy. The frozen wing was subsequently affixed to the device, and buzzy was positioned externally on the cheek area where the local anesthetic was to be administered. The local anesthetic was then administered through a conventional syringe in the vicinity of the tooth requiring the invasive treatment procedure. An experienced assistant, blinded to the procedural details, documented the objective parameter utilizing the Face, Legs, Activity, Cry, CONSOL ability while administration of the local anesthetic. Subsequently, following the deposition of the anesthetic solution, subjective parameters were assessed using the Wong-Baker Faces Pain Rating Scale.

## Statistical analysis:

Statistical analysis was performed on the collected data, with a significance level set at 5%. Data were analyzed to compare pain and discomfort levels between the two groups.

## Results:

Table 1 shows demographic details of the present clinical trial. There were 14 (46.6%) boys and 16 (53.4%) girls with mean age of 6.5±1.4 years. On intergroup comparisons, the differences in subjective and objective pain scores between the control and experimental groups were found to be statistically significant (Table 2).

## Discussion:

Intraoral administration of local anesthetics frequently constitutes one of the most distressing and anxiety-inducing aspects of pediatric dental care [5]. The apprehension surrounding the pain associated with these injections presents a significant obstacle to delivering effective dental treatment [6]. Ensuring adequate local anesthesia is imperative for the successful management of pediatric patients, as it significantly mitigates their anxiety and discomfort during various dental procedures [7]. To minimize the pain associated with local anesthetic injections, both pharmacological and non-pharmacological interventions have been explored. These methods include the use of topical anesthetics, reducing the rate of anesthetic infiltration, employing distraction techniques, vibrating the tissue surrounding the injection site during administration, and applying heat or cold prior to the injection. Among these, the application of flavored topical anesthetic gel is a prevalent practice in pediatric dentistry [7]. Anxiety related to dental visits and procedures is prevalent among pediatric patients, with age being a significant determinant of the severity of such anxiety. This study focused on children aged 6 to 10, as this age cohort typically possesses well-developed cognitive abilities. Buzzy® is an economically efficient and multifaceted plastic device, resembling a bee and equipped with cooled wings, that produces rapid vibrations [8]. The apparatus is a reusable, battery-powered, vibrating fish toy, incorporating dual 1.5-volt motors connected to a 9-watt battery [9,10]. The proposed mechanism of action is believed to correspond with gate control theory, which advocates that pain transmission from the peripheral nervous system to the central nervous system is regulated by a gating mechanism located in the dorsal horn of the spinal cord [11]. The vibratory component of Buzzy® activates the A-beta fibers (non-noxious motion nerves), thereby inhibiting the A-delta fibers (afferent pain receptors). Conversely, cold element stimulates the C fibers and, when applied before a painful stimulus, can also impede the A-delta pain signal. Empirical studies have demonstrated that Buzzy® surpasses placebos, vapocoolants, and analgesic creams in effectiveness [8, 10]. The WBFPRS was employed for the subjective assessment of pain, as it is regarded as a simple and effective scale for evaluating pain in young children [12]. Objective evaluation was done by the FLACC scale; it is a Behavioral Pain Rating Scale consists of distinct behavioral categories and various descriptors that consistently correlate with pain in children, adults with cognitive impairments, and individuals with critical illnesses, thereby corroborating the tool's validity within these populations [13]. This research evaluated the perception of pain in children assigned to either a group utilizing the Buzzy® device during the administration of a local anesthetic or a control group without the Buzzy® device. The findings indicate that the Buzzy® device constitutes an effective modality for mitigating pain perception during local anesthetic delivery. The finding of our study is aligned with those by Suohu *et al.* [4], Shetty *et al.* [8], Hegde *et al.* [9] and Subramaniam *et al.* [10].

## Conclusion:

Data shows that utilizing external cold and vibration via Buzzy® effectively reduces pain during administration of local anesthesia.

## Figures and Tables

**Table 1:** Demographics

**Gender**	**14 Boys (46.6%)**	**16 Females (53.4%)**	**Total= 30**
Age	Minimum 6	Maximum 10	Mean 6.5 ± 1.4

**Table 2:** Intergroup comparison of Clinical Variables

**Group**	**Mean Wong-Baker Faces Pain scale score**	**Mean FLACC score**
Group I Control	4.84 ± 1.50	4.75 ± 1.321
Group II Experimental	1.53 ± 1.02	1.67 ± 1.44
P value	< 0.01*	< 0.01*
*Significant
